# Silent Air Embolism of the Pulmonary Artery and Right Ventricle Due to Suspected Intravenous Substance Use Found Incidentally on Outpatient Imaging

**DOI:** 10.7759/cureus.114497

**Published:** 2026-08-13

**Authors:** Vivek Nayak M, Farage Ftiha, Saleem Shahzad

**Affiliations:** 1 Internal Medicine, NewYork-Presbyterian Brooklyn Methodist Hospital, Brooklyn, USA; 2 Pulmonary and Critical Care Medicine, NewYork-Presbyterian Brooklyn Methodist Hospital, Brooklyn, USA

**Keywords:** arterial air embolism, durant's maneuver, gas embolism, hyperoxygenation, intravenous drug user, pulmonary air embolism, vascular air embolism, venous air embolism

## Abstract

Pulmonary artery air embolism is a rare condition with significant morbidity and mortality. It most usually arises from iatrogenic entry of air into the venous system during surgeries and vascular access procedures. Most detected cases present with cardiopulmonary and neurological manifestations. It is exceedingly rare to find asymptomatic cases incidentally on imaging for other causes. Here, we describe the case of an elderly woman with ongoing chemotherapy sessions for her gynecologic malignancy, who was incidentally found to have pulmonary artery air embolism on an outpatient computed tomography (CT). The cause of this air embolism was suspected to be the unwitnessed episodes of intravenous drug use during her chemotherapy sessions. Although the patient had an asymptomatic and uncomplicated course, she was managed just as any symptomatic case would have been and safely discharged. This raises the point that an entity as dangerous as air embolism needs to be managed regardless of symptoms, in order to prevent all possible complications. This case highlights intravenous drug use as a potential non-iatrogenic cause of venous air embolism and also how some of them can be asymptomatic, yet warrant similar treatment as symptomatic ones.

## Introduction

Air embolism is a rare and potentially life-threatening condition in which air enters the vascular system and travels within it. It can be either venous or arterial, depending on where and how the air enters the bloodstream [1,2]. Venous air embolism is more common than arterial air embolism and occurs when air enters the venous system, ultimately reaching the right side of the heart, and then travels into the pulmonary arterial tree. This may be iatrogenic: during central or peripheral venous access, open or minimally invasive surgeries, and many other procedures. It can be trauma-induced as well [1,2]. Arterial air embolism is much rarer and usually occurs as a result of paradoxical embolism via right-to-left shunts [2].

Air embolism often presents with cardiopulmonary and cerebral manifestations, depending on the size and location of the air bubbles and their effects on the local hemodynamics [3-5]. Asymptomatic cases are seldom identified, yet they may be more common than we think. They are usually incidentally discovered on computed tomography (CT) while imaging for other reasons [6-8]. A systematic review estimates that among the identified cases of venous air embolism, less than 10% may be asymptomatic [9]. However, the actual numbers may be much higher. Intravenous drug use is a rare cause of venous air embolism, with a few cases reported in the literature [10-12]. Shah et al. describe a fatal case of massive venous air embolism causing cardiac arrest [10]. Mendelsohn et al. describe a case where more than 100cc of air was injected during drug use, which caused obstructive shock and stroke-like symptoms in the patient, eventually requiring catheter air embolectomy and hyperbaric oxygen therapy to save the patient's life [11]. However, there are other cases where air embolism was incidentally detected and not a cause of the presenting symptoms, such as the case by Brockman et al., in which a patient using intravenous cocaine was admitted for altered mental status and rhabdomyolysis-induced renal failure and CT had revealed large air bubbles within the right ventricle [12]. In this article, we describe a rare case of asymptomatic pulmonary artery air embolism suspected to be due to intravenous drug use, found incidentally on imaging. Our case differs from the above in that the patient was totally asymptomatic, without any derangements in vitals, and yet was carefully monitored and treated as any symptomatic case would have been.

## Case presentation

An elderly woman in her 70s, with recurrent stage IIIA1 uterine carcinosarcoma on chemotherapy, presented to the emergency department on the advice of her oncologist due to an abnormal CT finding of an "air bubble in her heart". She also had a history of type 2 diabetes, hypertension, chronic obstructive pulmonary disease (COPD), coronary artery disease, peripheral vascular disease, opioid use disorder (on methadone), diabetic retinopathy, diabetic foot ulcers, overactive bladder, and stage 4 chronic kidney disease (CKD).

The patient's uterine carcinosarcoma was diagnosed two years ago when she had developed abnormal uterine bleeding. She had undergone a complex surgery involving total radical hysterectomy (TRH), bilateral salpingo-oophorectomy (BSO), pelvic and para-aortic lymphadenectomy (PPALND), and omentectomy the same year and was lost to care until a few months back, at which time a mass was palpated on pelvic exam. Positron emission tomography-CT (PET-CT) had demonstrated numerous new metastases, especially to lymph nodes in the thorax and abdomen. A biopsy of the abdominal wall metastasis was performed, which demonstrated disease progression. The patient was planned to be restarted on chemotherapy (carboplatin + paclitaxel + dostarlimab) and also planned for a CT of the chest, abdomen, and pelvis before the next follow-up visit. She was unable to receive regular outpatient chemotherapy successfully. Several incidents had been reported by an infusion nurse at the infusion center, where the patient seemed to be fine until she had prolonged bathroom breaks, after which she became poorly responsive and obtunded, with respiratory depression and pinpoint pupils, and needed to be sent to the emergency room repeatedly. On a few occasions, administration of intranasal naloxone had improved the patient's mentation and respiration. Urine toxicology screen was positive for cocaine and opiates in one of the emergency visits. However, substance use was never directly witnessed, and the patient endorsed that she was not using. The patient eventually got a CT of the chest, abdomen, and pelvis outpatient as planned, which revealed the progression of disease and also a new finding of moderate air embolism of the pulmonary artery and right ventricle.

The patient presented to the emergency department at the advice of her outpatient oncologist after the air embolism was found on the outpatient CT. She reported she was at her baseline. She did not have any dyspnea, chest pain, or palpitations. She had a chronic non-productive cough that had not changed in character, with no hemoptysis. She denied any recent intravenous drug use. Vital signs were stable, and physical examination was largely unremarkable.

The patient was admitted to the medical stepdown unit for the management of pulmonary artery air embolism. While the source was unknown, it may most likely be from one of three routes: first, from unreported intravenous drug self-injection; second, from erroneous intravenous cannulation for outpatient chemotherapy; and third, from erroneous intravenous contrast injection prior to CT. The first route seems the most likely. The patient's many episodes of obtundation post-bathroom visits during outpatient chemotherapy suggest the possibility of intravenous drug use by the patient, possibly via the erroneous manipulation of the infusion cannula, that may have caused venous air embolism. Intravenous cannulation as a route for venous air entry seems less likely, as this was in a tertiary cancer care center with well-trained nurses who insert intravenous lines per protocol. The third route as well seems less likely as the CT was done in the same hospital and intravenous contrast injection protocols are strictly followed here.

**Figure 1:**
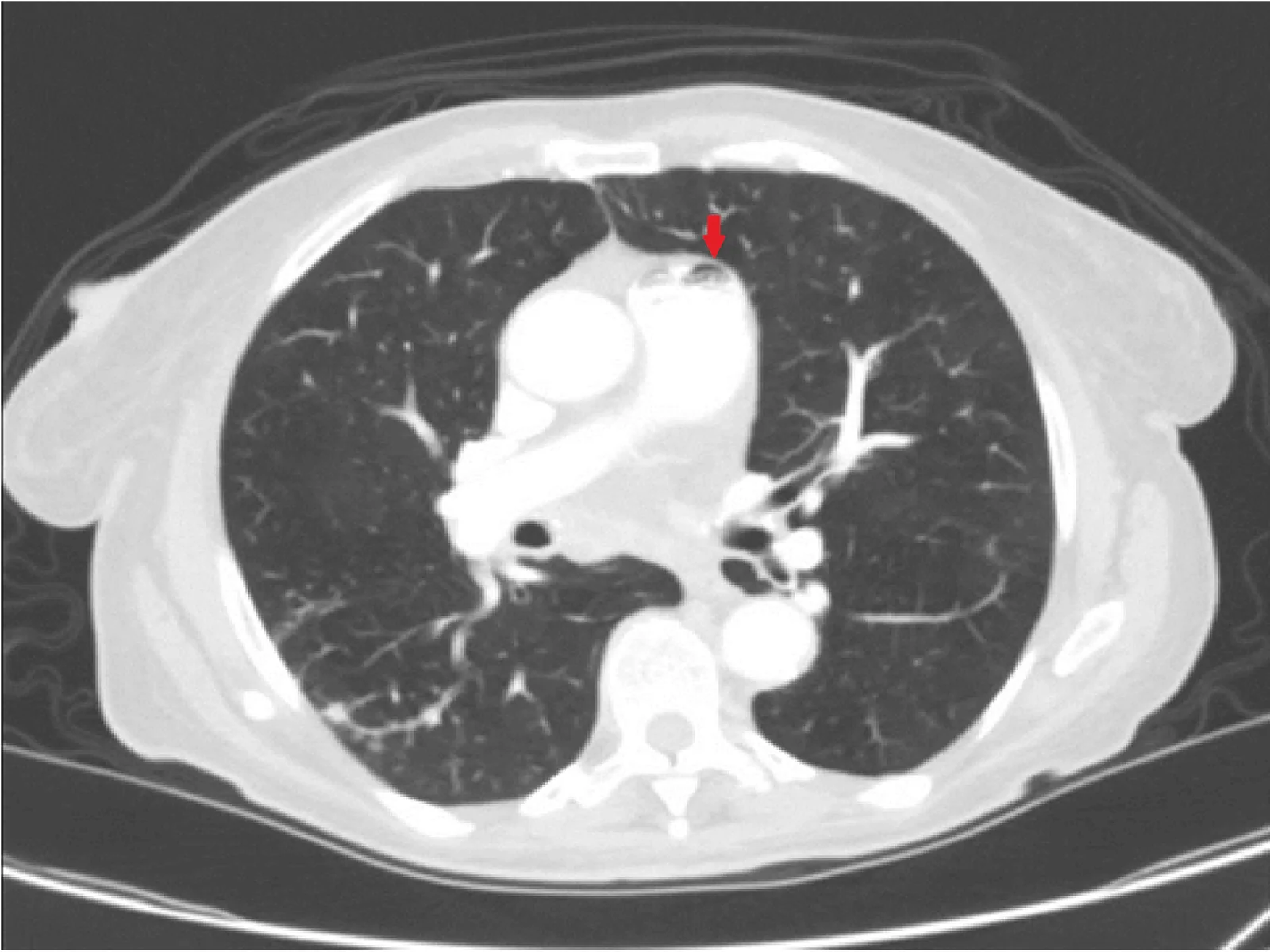
CT of the chest axial view Moderate air embolism within the main pulmonary artery and right ventricle, in the form of air bubbles of various sizes (red arrow). CT: computed tomography

Initial laboratory investigations were notable for hyponatremia, hypochloremia, acute kidney injury (AKI) on CKD (serum creatinine 2.71 mg/dL, baseline 1.2-1.5), and mild respiratory alkalosis. Electrocardiogram revealed normal sinus rhythm, with P-mitrale and P-pulmonale patterns suggestive of biatrial enlargement, and occasional premature ventricular complexes. The CT of the chest (Figure 1) done recently had shown moderate air embolism in the main pulmonary artery and right ventricle, in addition to bilateral pulmonary metastatic nodules and multi-station thoracic lymphadenopathy. A bilateral lower extremity venous duplex scan did not reveal any deep vein thrombosis.

**Figure 2:**
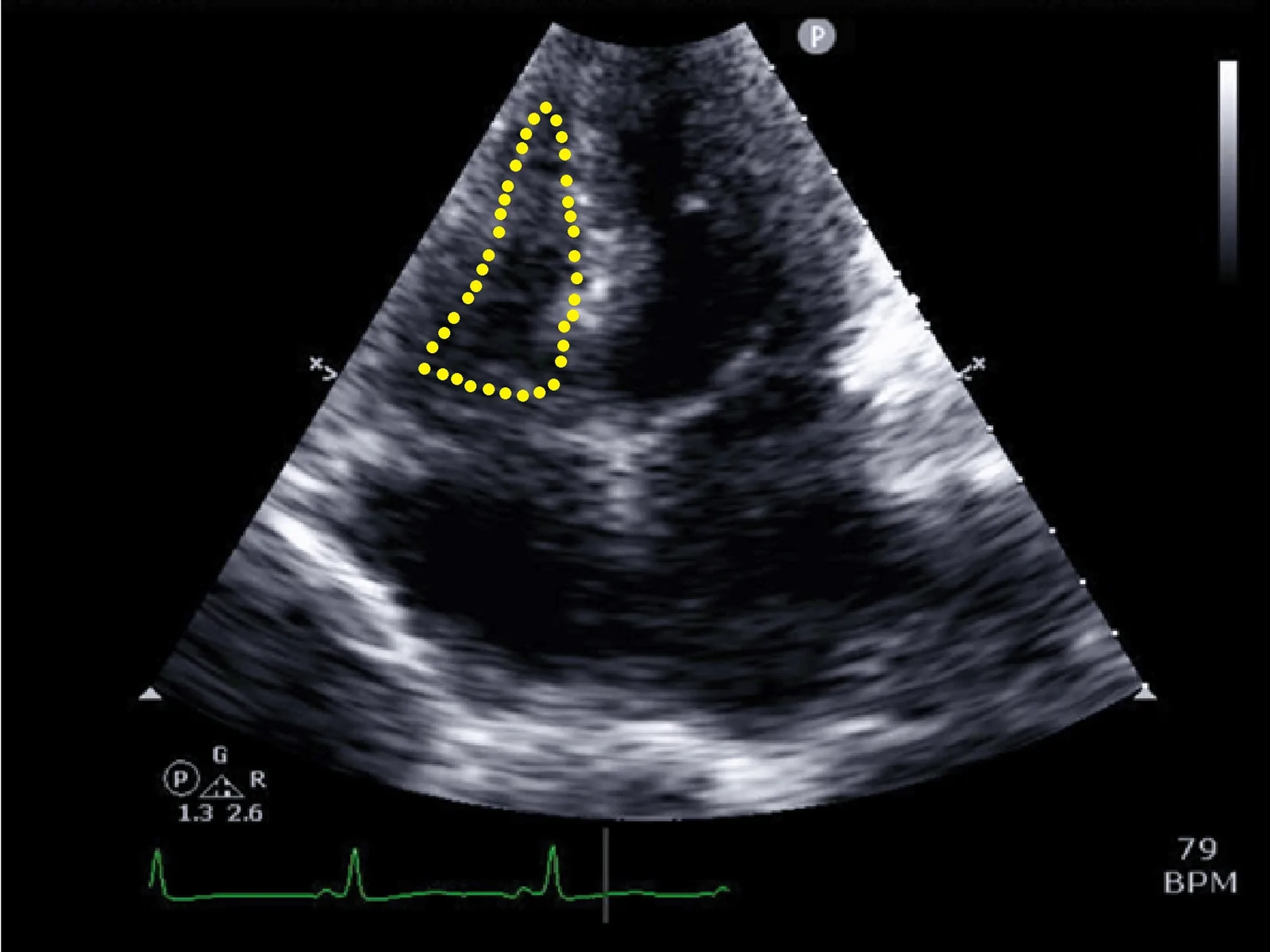
Two-dimensional transthoracic echocardiography Apical four-chamber view showing air bubbles as numerous hyperechoic foci within the right ventricle cavity (roughly outlined by a yellow dotted line), as opposed to the anechoic nature of the other heart chambers.

A transthoracic echocardiogram (Figure 2) revealed a normally sized left ventricle, with the wall thickness mildly to moderately increased and the left ventricular ejection fraction estimated to be 60-65%. Grade I left ventricular diastolic dysfunction was present with normal filling pressures. The right ventricle was normal in size and function and demonstrated multiple hyperechoic mobile foci, likely air bubbles from the embolism. The left atrium was mildly dilated, while the right atrium was normal in size. Pulmonary artery systolic pressure was normal. The inferior vena cava was normal in size with normal inspiratory variations, consistent with normal right atrial pressure. No pericardial effusion was seen.

Although the patient was asymptomatic, she was placed on a high-flow nasal cannula with 100% fraction of inspired oxygen (FiO2) at 45 L/min. She was maintained in the left lateral decubitus position with head down (Durant's position). The rationale behind the steps was to (1) have the air bubbles dissolve as soon as possible and (2) prevent their travel further distally along the pulmonary arterial tree. These were mainly preventative measures done to avoid any clinical decompensation in the patient, highlighting the role of defensive medicine in treating potentially dangerous conditions when they present in a benign way. These measures were in place for at least 48 hours.

The patient remained hemodynamically stable and asymptomatic. Hence, she was weaned to room air. Ipratropium and albuterol nebulizations were given as needed for COPD. Given no concerning signs of right ventricular strain on the echocardiogram, the patient was deemed medically stable for discharge and was encouraged to lie in Durant's position at home while resting.

Subsequently, the patient had admissions for inpatient chemotherapy sessions, given concern for covert intravenous drug use during the outpatient chemotherapy sessions. During these admissions, the serum creatinine level had returned to baseline. The previous AKI was likely due to intravenous contrast. The patient has not undergone a repeat CT of the chest to demonstrate resolution of the air embolus, given that the risk of repeat AKI outweighs the benefit of demonstrating resolution. This is one of the major limitations of this case as radiologic resolution of air embolism was not demonstrated and the patient was discharged without this confirmation. Given the moderate size of the air embolism, the absence of clinical manifestations, and >48 hours of high-flow oxygen therapy, it was just deemed that the air embolism must have resolved.

## Discussion

An air embolus is a bubble of air that lodges inside a blood vessel, potentially stopping blood flow [13]. Air embolism falls under the larger umbrella of gas embolism and is the most common form; however, specific gas embolisms do occur, such as those involving carbon dioxide, nitrogen, and nitrous oxide [2]. Venous air embolism is the most common type of air embolism. Venous air entry is greatly facilitated by the negative (sub-atmospheric) pressure within the veins and the tendency of some veins to be non-collapsible, such that they do not close shut on being cut, leaving a gateway for air entry [2,3]. Venous air embolism has a wide range of causes, from iatrogenic to traumatic [1]. Arterial air embolism is relatively rare, although it can be more severe. It occurs most often as a result of paradoxical embolism via right-to-left shunts. Less common causes include decompression barotrauma and extracorporeal bypass during cardiac surgeries. Even small air bubbles in the arterial system can be deadly if they end up within the coronaries or cerebral arteries, causing abrupt ischemia and infarction [2].

Venous air embolisms are mostly iatrogenic and can occur as a consequence of any procedure involving venous access, including central venous catheterization, peripheral vein cannulation, and intravenous injections. They can also occur during open surgeries, especially neurosurgical procedures and head and neck surgeries, as many of the veins cut in these may not easily close shut as they are held open by fascial planes that they penetrate and the negative pressure within sucks the air in [1,2]. According to a systematic review by Alzghoul et al. [9], the most common cause of iatrogenic air embolism was surgery, followed by central venous catheterization, peripheral venous cannulation, and ventilator barotrauma. The literature also describes cases of iatrogenic pulmonary air embolism due to inadvertent venous air entry during contrast injections for contrast-enhanced CT [3-5]. Invasive procedures involving the lung parenchyma, such as biopsies or local ablations, can create broncho-vascular fistulae that lead to air embolism [7]. Apart from iatrogenic causes, there are traumatic causes such as gunshot wounds, sharp weapon trauma, and rib fractures [9]. Intravenous drug use is a rare cause of venous air embolism [10-12]. They may occur with peripheral venous injections, but also with direct injections into the central veins in the neck, the so-called "pocket shots". Its presentation can range from asymptomatic cases, such as this patient, to severely symptomatic cases as described prior in the introduction.

The entry of a significant amount of air into the right side of the heart, and further into the pulmonary arterial circulation, acts similarly to that of a thrombus, causing distal obstruction to pulmonary circulation, elevated pulmonary artery and right heart pressures, and strain on the right heart [2]. A large amount of air in the right ventricle can cause an "air-lock" effect, leading to ineffective right ventricular contraction and acute right heart failure [5]. In the lungs, it causes increased intrapulmonary right-to-left shunting, ventilation-perfusion mismatch, and increased alveolar dead space, ultimately causing hypoxia [1]. Hypoxia can cause pulmonary vasoconstriction and acute pulmonary hypertension [5]. Air embolism also causes decreased preload to the left ventricle, which can result in an obstructive shock-like picture. Altered cardiac pressures can trigger tachyarrhythmias. Causes of death include circulatory collapse, acute cor pulmonale, or arrhythmias leading to cardiac arrest [2]. Apart from obstructive pathophysiology, the air bubbles can cause vascular endothelial shear stress and can trigger vascular inflammation, which may manifest as pulmonary edema and bronchospasm [1].

Sometimes these air bubbles can paradoxically embolize into the arterial system via right-to-left shunts such as a patent foramen ovale (PFO). When venous air embolism is significant, it can often pass through pulmonary circulation into the left heart and then embolize arterially. Once air bubbles enter the left heart, they can terminate in any vessel anywhere in the body. Only coronary and cerebral arterial air embolism can cause catastrophic consequences. Coronary air embolism can cause obstruction of end arteries, myocardial ischemia, and infarction. Cerebral arterial embolism can cause injury to brain tissue by two mechanisms: first, obstruction of blood flow, ischemia, and infarction and, second, endothelial damage, causing local inflammation. These may ultimately cause vasogenic edema and trigger seizures [2]. The air bubbles may eventually diffuse across vascular walls into the alveolar air and are exhaled into atmospheric air, resolving the embolism [3]. However, they may leave behind permanent ischemic damage to tissues before dissolution.

Patients with air embolism usually present either with cardiopulmonary symptoms like dyspnea, chest pain, cough, palpitations, or cyanosis or with neurological symptoms like weakness, seizures, or headaches, depending on where the air bubbles have lodged [1,9]. The classic auscultatory finding of systolic "mill-wheel murmur" is only heard when there is a significant amount of air in the heart [2]. However, when the amount of air is minimal, there may be no signs or symptoms at all. Asymptomatic cases of air embolism have been described, and these have often been found on immediate post-procedure imaging studies or during contrast imaging studies [6-8].

The simplest and quickest way to identify intracardiac air is transthoracic echocardiography, supported by color Doppler. Air bubbles are seen as hyperechoic foci [2]. However, transesophageal echocardiography is a more sensitive sonographic mode for detection [2]. Often, large intracardiac air emboli can be seen on chest radiographs as lucencies [1]. Contrast-enhanced CT is the easiest and most definitive diagnostic method. Venous air embolism is seen as air bubbles within the right side of the heart, pulmonary arteries, vena cavae, or other vessels. Sometimes, due to motion artefact, these air bubbles can demonstrate a tri-radiate appearance, the so-called "dynamic Mercedes Benz sign" [4].

Other investigations include an electrocardiogram, which may show ischemic ST-T if the coronaries are involved or may also show changes of right heart strain if it is a massive pulmonary air embolus, such as P-pulmonale, suggesting right atrial enlargement [2]. End-tidal carbon dioxide (EtCO2) measurement is a sensitive and rapid diagnostic method, although not very specific. A sudden drop in EtCO2 in a patient undergoing surgery under general anesthesia and mechanical ventilation may signal air embolism, enabling the early recognition of air emboli during surgery [9]. Arterial blood gas analyses may reveal hypoxemia and respiratory acidosis and, in cases of advanced tissue hypoperfusion, even metabolic/lactic acidosis [9].

Venous air embolism is a cause of significant morbidity with frequent intensive care unit (ICU) admissions and a need for life support. Its all-cause mortality is estimated at around 18.4% based on a systematic review by Alzghoul et al., which included all patients admitted to a hospital with a diagnosis of venous air embolism, whether symptomatic or not [9]. Therefore, expedited treatment is essential. The most important, urgent, and rapidly achievable step in treatment is active repositioning of the patient to prevent the migration of air bubbles into the pulmonary circulation. This is achieved by maintaining the patient in the left-lateral decubitus position, with the head down (Trendelenburg position), and this combination is called Durant's maneuver [4,14]. This allows the air bubbles to remain in the apex of the right ventricle and prevents their access to the right ventricular outflow tract [4]. Definitive management of air embolism lies in enhancing the dissolution of the air and decreasing the size of the air bubbles. This is achieved by hyperoxygenation. Supplying the patient with 100% oxygen creates a gradient for nitrogen (which occupies most of the bubble volume) to diffuse out of the air bubbles, thereby decreasing their size. This may be achieved by various means of oxygenation, ranging from high-flow nasal cannula to mechanical ventilation. In severe cases, hyperbaric oxygen therapy may be necessary, and it accelerates the process of nitrogen dissolution. It also supplies the tissues with high oxygen to counteract the effects of ischemia. Hyperbaric oxygen treatment is the first-line therapy for arterial air embolism. It is also important to prevent further entry of air into the venous system, which can be achieved by increasing venous pressure (making it less negative) through aggressive volume resuscitation and the Valsalva maneuver [2,15]. As a last resort, a central venous catheter or pulmonary artery (Swan-Ganz) catheter can be used to aspirate the air from the right heart or pulmonary artery [2,4]. This is called air embolectomy [10]. Ventilatory support, inotropes, and cardiopulmonary resuscitation may be necessary in severe cases with cardiovascular compromise [2]. External cardiac compression or massage may facilitate the dispersion of a large air embolus into smaller bubbles, relieving circulatory strain [4,5].

Depending on the size of the air embolus and the presenting symptoms, the management may differ. Small asymptomatic cases may just be monitored on room air as there is little evidence supporting aggressive treatment in such cases. However, moderate-to-large and symptomatic cases are definitely treated aggressively. However, in patients with right-to-left intracardiac shunts or pulmonary arteriovenous connections, paradoxical embolism may occur even with a small venous air embolus, potentially leading to cerebral infarction and neurological deficit; hence, such patients need to be treated aggressively as well [16]. A PFO must always be excluded in patients with venous air embolism. In our patient, although PFO was excluded on transthoracic echocardiogram, she still had a risk of paradoxical embolism given that her pulmonary metastatic nodules were a potential source of arteriovenous connection and transfer of air bubbles to the left side of the heart [17]. Hence, she was treated aggressively with available non-invasive means like high-flow oxygenation and Durant's position, despite no symptoms and only a moderate-sized air embolism on imaging. This mostly had a preventative and defensive intention.

## Conclusions

Although air embolism can be serious and life-threatening, asymptomatic and uncomplicated cases do occur. Intravenous drug use is a rare yet potential cause of air embolism. The size of the air embolus and the location where it lodges ultimately determine the severity and clinical manifestations of air embolism. The basic framework of treating air embolism remains the same regardless of the clinical presentation and includes Durant's maneuver and hyperoxygenation. While large and symptomatic air embolisms need aggressive treatment, small asymptomatic ones may be monitored without it. However, small asymptomatic air embolisms need to be treated aggressively when they have a high risk of paradoxically embolizing into the arterial system, where they can have catastrophic consequences regardless of size. These risks for paradoxical embolism include intracardiac shunts like PFOs and intrapulmonary shunts such as arteriovenous malformations and lung malignancies, including metastases. Overall, each case of venous air embolism needs a tailored risk-benefit discussion before initiating treatment, and the intent of treatment should be well defined, whether curative or preventative.
